# Multisource Synergistic Electrocatalytic Oxidation Effect of Strongly Coupled PdM (M = Sn, Pb)/N-doped Graphene Nanocomposite on Small Organic Molecules

**DOI:** 10.1038/srep14173

**Published:** 2015-10-05

**Authors:** Peng Wu, Yiyin Huang, Longtian Kang, Maoxiang Wu, Yaobing Wang

**Affiliations:** 1 State Key Laboratory of Structural Chemistry; Key Laboratory of design and assembly of functional nanostructures, Fujian Institute of Research on the Structure of Matter, Chinese Academy of Sciences, YangQiao West Road 155#, Fuzhou, 350002, P. R. China

## Abstract

A series of palladium-based catalysts of metal alloying (Sn, Pb) and/or (N-doped) graphene support with regular enhanced electrocatalytic activity were investigated. The peak current density (118.05 mA cm^−2^) of PdSn/NG is higher than the sum current density (45.63 + 47.59 mA cm^−2^) of Pd/NG and PdSn/G. It reveals a synergistic electrocatalytic oxidation effect in PdSn/N-doped graphene Nanocomposite. Extend experiments show this multisource synergetic catalytic effect of metal alloying and N-doped graphene support in one catalyst on small organic molecule (methanol, ethanol and Ethylene glycol) oxidation is universal in PdM(M = Sn, Pb)/NG catalysts. Further, The high dispersion of small nanoparticles, the altered electron structure and Pd(0)/Pd(II) ratio of Pd in catalysts induced by strong coupled the metal alloying and N-doped graphene are responsible for the multisource synergistic catalytic effect in PdM(M = Sn, Pb) /NG catalysts. Finally, the catalytic durability and stability are also greatly improved.

The rapid increase in energy demands, greenhouse gas emissions and depletion of fossil fuels make unquestionably direct alcohols fuel cells (DAFCs) attractive technology due to highly efficient fuel utilization and environmentally-friendly operations^1^,^2^,^3^,^4^,^5^. In the commercial adoption of DAFCs, anode catalysts with low activity and durability are the bottleneck for high performance fuel cells, as they make up a substantial part of the cost and performance of the cells^2^. Although oxidation of small organic molecules such as alcohols (methanol, ethanol, ethylene glycol etc.) possesses distinct advantages owing to its high energy density, low toxicity, easy storage and transportation, and large scale of production from biomass and chemical industry. Some poisonous intermediates, such as CO, will be generated and strongly adsorbed on catalyst surface in the process of electro-oxidation^6^,^7^,^8^, blocking the surface active sites from further catalysis. This results in a dramatic decrease in electrochemical activity of the catalyst. However, the development of catalyst with high activity and high tolerance towards CO-like specie poisoning is still restricted. Therefore, careful electrocatalyst design strategies must be applied to strengthen the activity and anti-poisoning issues associated with both the catalyst metal particles and carbon supports to achieve practical adoption targets.

Recently, to realize the practical adoption of palladium (Pd)-based catalysts, great research efforts have been devoted to introducing a second element such as Pt, Au, Sn, Ni, Cu, Bi, W^9^,^10^,^11^,^12^,^13^,^14^,^15^,^16^ to Pd supported on carbon for optimization of poisoning tolerance and electrocatalytic performance of Pd toward small organic molecule oxidation in alkaline media based on the bimetallic synergistic effect. The anti-poisoning and activity of Pd-based catalyst can be further improved by growing the nanoparticles onto novel nanostructured support materials, such as graphene and carbon nanotube^17^,^18^, unlike the conventional carbon materials^19^,^20^. Graphene in particular possesses unique chemical, mechanical, and electrical properties as catalyst support. Studies have additionally shown that graphene doped with heteroatoms is an effective way to tune the intrinsic properties, due to the substitutional heteroatom sites might provide the main initial nucleation sites for the formation of noble metal nanoparticles and also enhance the interaction with nanoparticles and improve the electrocatalytic activity^21^,^22^,^23^. In our previous work, we demonstrated that N-doped graphene (NG) is a highly promising support to load Pd nanoparticles for enhanced activities^24^, Zhu *et al*. has reported the similar work in which improved electrocatalytic activity for ethanol oxidation was obtained from Pd@N-doped carbon^25^. To our best knowledge, compared with single metal doping or altering support carbon materials to improve catalytic performance, the research on joint of metal alloying and altered support material in one catatlyst for synergetic electrocatalysis is still limited.

Herein, a series of palladium-based catalysts of metal alloying (Sn, Pb) and/or (N-doped) graphene support with regular enhanced electrocatalytic activity were investigated. We employed PdSn/NG as a typical catalyst to investigate the metal alloy and N-doped graphene support effect on the activity and anti-poisoning during ethylene glycol oxidation. By combining the advantages of both the metal alloying and unique N-doped graphene support materials, it can not only form a uniform nanoparticle dispersion, but also create a unique electronic structure and high Pd(0)/Pd(II) ratio of Pd-based catalysts. This effect makes the Pd-based catalysts much more catalytically active and better poisoning-tolerant than the sum of singly doped Pd-based catalysts. Furthermore, it is demonstrated in the extend experiment that this multisource synergetic effect should be universal in the PdM(M = Sn, Pb)/NG catalyst toward small organic molecule oxidation.

## Results and Discussion

The presence of Pd and Sn in the catalysts was verified by ICP analysis. The practical Pd/Sn loadings in Pd/G, Pd/NG, PdSn(2:1)/G and PdSn(2:1)/NG are 17.6 wt.%, 16.3 wt.%, 17.4 wt.%/9.5 wt.%, 15.8 wt.%/8.7 wt.%, and the practical atomic ratios of Pd:Sn in PdSn/G and PdSn/NG were 8:3.91 and 8:3.95, respectively. It confirms that the catalysts are successfully synthesized, while it should be noted that the practical atom contents of Sn are slight lower than its initial added amount in the synthesis process because Sn was not completely reduced and deposited by NaBH_4_^24^.

The XRD patterns of Pd/G, Pd/NG, PdSn(2:1)/G and PdSn(2:1)/NG catalysts are shown in Figure S1. The first broad peak at 26.3° refers to C (0 0 2) facet of graphite. The other four diffraction peaks of the catalysts at 39.8°, 46.4°, 67.7° and 80.6°, are corresponding to (1 1 1), (2 0 0), (2 2 0) and (3 1 1) planes of the Pd fcc crystal (JCPDS-ICDD, Card No. 65–6174), respectively. The half peak width of Pd/NG at 39.8° is ca. 1.33°, broader than that of Pd/G (ca. 0.69°), and the one of PdSn(2:1)/NG at 39.8° is ca. 1.32°, broader than that of PdSn(2:1)/G (ca. 0.70°). This phenomenon is probably caused by the smaller size of Pd and PdSn in N-doped graphene than in graphene support, which may be favorable for catalysts activities based on more active sites^24^,^25^. While no obvious peaks related to Sn and/or its oxides are found in the PdSn/G and PdSn/NG catalysts, it may exist as thin and/or amorphous phases on the catalyst surface^26^,^27^. The addition of Sn causes clear negative shifts of the Pd peaks for these catalysts, indicating parts of Sn atoms are alloyed with Pd^12^.

**Figure 1:**
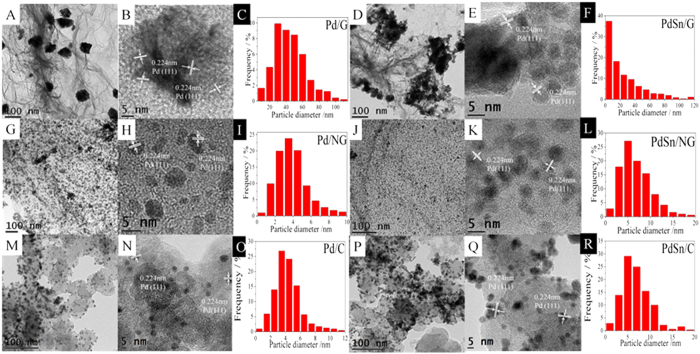
TEM images of Pd/G(**A**,**B**), Pd/NG (**G**,**H**), PdSn/G (**D**,**E**), PdSn/NG (**J**,**K**), Pd/C (**M**,**N**) and PdSn/C (**P**,**Q**) and the particle size distribution of Pd/G (**C**), Pd/NG (**I**), PdSn/G (**F**), PdSn/NG (**L**), Pd/C (**O**) and PdSn/C (**R**).

The dispersion state of the as-prepared Pd/G (A/B), Pd/NG (G/H), PdSn/G (D/E), PdSn/NG (J/K), Pd/C (M/N) and PdSn/C (P/Q) catalysts is displayed in Fig. 1. The corresponding particle size distribution based on the statistics of 500 particles is displayed in Fig. 1C,F,I,L,O,R. As shown in Fig. 1A,C, the dispersion of synthesized Pd nanoparticles on graphene is nonuniform with a wide diameter range of 2 ~ 100 nm. It is shown that the large particles are the aggregation of small ones, which is confirmed by different direction of lattice fringes Pd (111) in one particle as shown in Fig. 1B. In contrast, the Pd nanoparticles are uniformly dispersed on N-doped graphene surface with only a little aggregation in Pd/NG as shown in Fig. 1G,H. Most nanoparticles on N-doped graphene are in the diameter range of 2 ~ 10 nm as shown in Fig. 1I. The dispersion of synthesized PdSn nanoparticles on graphene is also nonuniform with a wide diameter range of 2 ~ 120 nm similar to Pd nanoparticles as shown in Fig. 1D,E,F. In contrast, the PdSn nanoparticles are uniformly dispersed on N-doped graphene surface with only a little aggregation similar to Pd/NG as shown in Fig. 1J,K. Most nanoparticles on PdSn/NG are in the diameter range of 2 ~ 20 nm as shown in Fig. 1L. These results imply that the N-doped graphene facilitates the nanoparticle uniform dispersion on the supports. The uniform dispersion of metal nanoparticles on N-doped graphene is caused by N-based groups of bulge on graphene surface, which acts as anchor sites for nucleation of metal ions and thus finally induces uniform deposition of metal nanoparticles^28^,^29^,^30^. The uniform dispersion of metal nanoparticles supported on N-doped graphene is the basis for high performance of the catalyst^12^. Furthermore, this N-doping can also enhance the electrical properties of graphene, and affords the active sites of the catalyst with a charge polarization effect^31^,^32^. Compare the NG supported catalysts (Pd/NG, PdSn/NG) with their amorphous carbon supported counterparts (Pd/C, PdSn/C), metal nanoparticles have similar average diameteras displayed in Fig. 1M–R. Thus performance difference derived from particle size effect can be eliminated.

The energy-dispersive X-ray spectroscopy (EDS) mapping profile shown in Figure S2 obviously manifested the homogenous distribution of nitrogen, carbon, tin and palladium in the PdSn/NG catalyst. This result is in good agreement with TEM (Fig. 1) observations. The uniform distribution of nitrogen provides a uniform anchor sites for metal deposition and the uniform distribution of tin could promote the catalytic activity of Pd catalyst.

**Figure 2:**
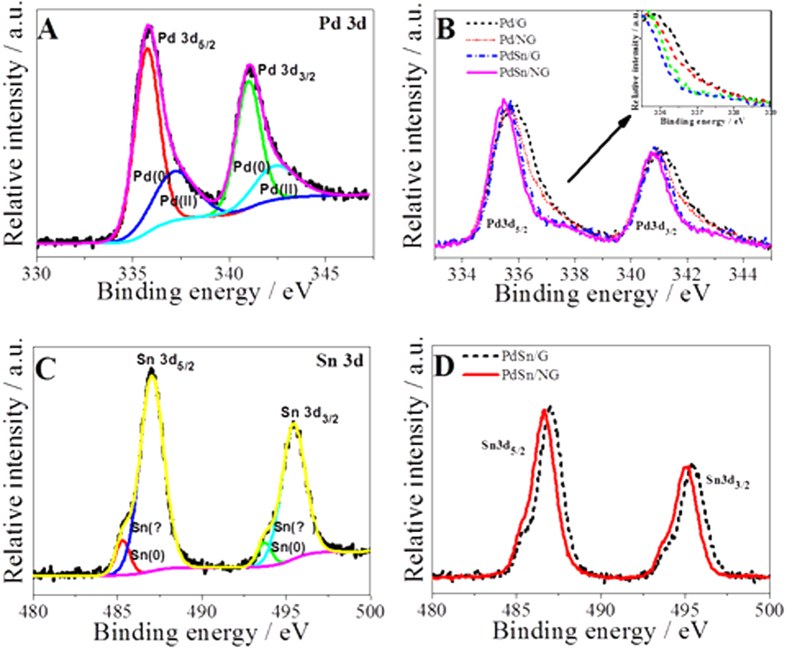
(**A**) XPS spectrum of Pd/G in Pd 3d region; (**B**) XPS spectrum of Pd/G, Pd/NG, PdSn/G and PdSn/NG in Pd 3d region; (**C**) XPS spectra of PdSn/G in Sn3d region; (**D**) XPS spectrum of PdSn/G and PdSn/NG in Sn3d region.

The valence states and their interaction of Pd, Sn, N, O and C in the catalysts were determined by the XPS analysis, as shown in Fig. 2 and Fig. S3. Two pairs of asymmetric peaks constitute the Pd 3d signal in Fig. 2A. The binding energies (BEs) of Pd 3d_5/2_ (335.8 and 337.3 eV) are 5.2 eV lower than those of Pd 3d_3/2_ (341.0 and 342.5 eV) for each doublet. The intense doublet peaks belong to Pd (0) and the weak peaks are attributed to Pd (II) species, such as PdO and Pd(OH)_2_^30^, the ratio between bivalent species and metallic Pd is 0.20:1. Two pairs of asymmetric peaks constitute the Sn 3d signal in Fig. 2C. The binding energies (BEs) of Sn 3d_5/2_ (487.1 and 485.3 eV) are 6.6 eV lower than those of Sn 3d_3/2_ (485.3 and 495.5 eV) for each doublet. The weak doublet peaks belong to Sn (0) and the intense peaks are attributed to Sn (IV) species, such as SnO_2_ and Sn(OH)_4_^12^, the ratio between metallic Sn and tetravalent species is 0.07:1. Existence of more tetravalent Sn is attributed to its very easy oxidation as exposed in air. Both Sn and its oxides could play an important role in the electro-catalytic oxidation of small organic molecules because they may facilitate adsorption of oxygenated species and oxidation of poisonous species^12^,^19^. The change of Pd 3d signal in different environment is shown in Fig. 2B. It is found the Pd 3d_5/2_ peaks of Pd/NG and PdSn/G shift negatively about 0.1 eV compared to that of Pd/G, and the peak of PdSn/NG further shifts negatively about 0.2 eV compared to that of Pd/NG and PdSn/G as shown in Table S1. This conformed the electron coupling of Pd by Sn and N-doped graphene respectively, which is because Sn and N in the support have low electronegativity compared to Pd and they can increase the electron cloud density of Pd. Further, it should be note that the bivalent Pd species in PdSn/G and PdSn/NG is lower than that in Pd/G and Pd/NG as show in the insert of Fig. 2B, which implies that Sn has a function in suppressing formation of the bivalent species of Pd in the catalyst. This is important to improve the catalyst performance^30^. Moreover, the comparison of Sn 3d between PdSn/G and PdSn/NG is shown in Fig. 2D. It is found the Sn 3d peak of PdSn/NG shifts negatively compared to that of PdSn/G, this further confirms that the PdSn alloying coupling with N-doped graphene, which is because N atom of the doped graphene has high electronegativity compared to Sn and they can increase the electron cloud density of Sn. This electronic interaction may play an important role in catalytic reactions. The electron cloud density of Pd was altered simultaneously by N-doped graphene supporting and alloyed Sn. Such superimposed action yielded a synergetic effect, which can improve the catalytic activity of Pd catalyst to the utmost^16^.

**Figure 3:**
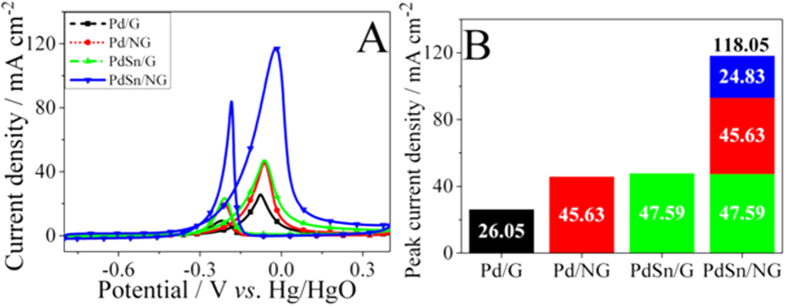
(**A**) Cyclic voltammograms of Pd/G, Pd/NG, PdSn/G and PdSn/NG in the 1 M KOH + 0.5 M (CH_2_OH)_2_ solutions. Scan rate: 50 mV s^–1^; (**B**) the peak current density of different catalysts.

**Figure 4:**
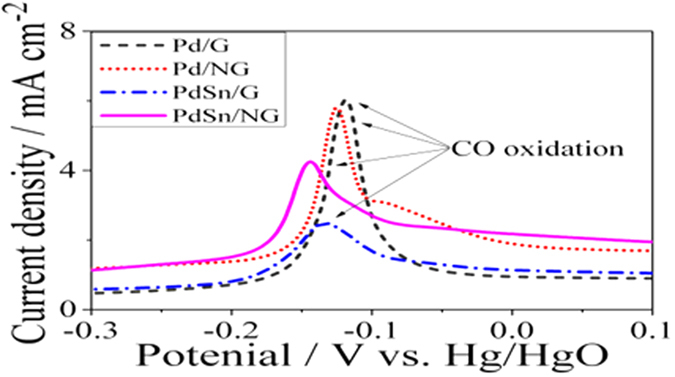
CO stripping curves on Pd/G, Pd/NG, PdSn/NG and PdSn/NG in 1 M KOH solution. Scan rate: 50 mV s^−1^.

**Figure 5:**
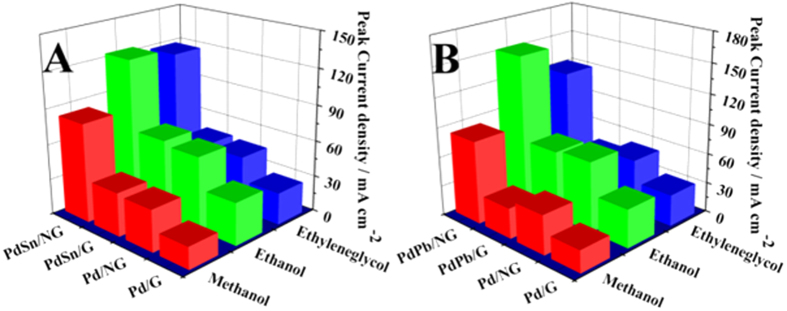
The cyclic voltammograms of the series of other Pd-based catalyst with metal alloying and/or (N-doped) graphene in the 1 M KOH + 1 M Methanol/Ethanol/Ethylene glycol solution.

All catalysts were loaded onto a glassy carbon electrode for comprehensive evaluation of their catalytic performance, as shown in Figs 3, 4, 5 and Figs S4–S14. The ethylene glycol oxidation reaction (EGOR) on the as-prepared catalysts was performed by using cyclic voltammetry in 1 M KOH + 0.5 M (CH_2_OH)_2_ solution. The curves were recorded after a stable response is obtained and the current was normalized with respect to the electrode area. The CV curves of PdSn/NG with different Pd/Sn atomic ratios are exhibited in Fig. S4. The current density of anode oxidation peak at ca. –0.03 V firstly increased from 65.93 to 118.05 mA cm^−2^ and then fall down to 98.85 mA cm^−2^ with the increasing Sn content in the catalyst, while the peak potential shifts positively from –0.05 to –0.02 V. The PdSn(2:1)/NG catalyst possesses the highest current density of 118.05 mA cm^−2^ at the potential of –0.03 V. This result suggests that adding Sn could promote the Pd catalytic activity in ethylene glycol oxidation reaction; but excessive Sn probably leads to exceeded coverage of Pd active sites^24^. Thus the catalyst activity for ethylene glycol electrooxidation achieves the best as Pd/Sn atomic ratio is 2:1. The currents for EGOR on the Pd/G, Pd/NG, PdSn/G and PdSn/NG catalysts are 26.05, 45.63, 47.59 and 118.05 mA cm^−2^, respectively, as shown in Fig. 3. It is noted that the activity of the Pd/NG and PdSn/G is higher that of the Pd/G, the higher activity of Pd/NG is resulted from both the electronic effect as well as the smaller and more uniform metal nanoparticles on N-doped graphene than on graphene, while that of PdSn/G is mainly originated from the optimized electron cloud density and valence state of Pd. Surprisingly, the peak current density (118.05 mA cm^−2^) of PdSn/NG is higher than the sum of peak current densities (45.63 + 47.59 mA cm^−2^) of Pd/NG and PdSn/G. It reveals a synergistically electrocatalytic oxidation effect exists in PdSn/N-doped graphene nanocomposite, this agrees with the analyses of the XPS data.

Stable cyclic voltammograms (CVs) in a 1 M KOH solution are recorded to detect the surface state of the as-prepared catalysts. As presented in Figure S5, broad peaks between –0.90 to –0.60 V may be ascribed to the hydrogen desorption/adsorption regions of the catalysts. The reduction peaks for oxygenated species are located at ca. –0.22 V, which is similar to the result of previous reports^33^,^34^,^35^. The double electrode layer capacitance of Pd/NG and PdSn/NG is much higher than Pd/G and PdSn/G, respectively, which may benefit ethylene glycol dehydrogenation^35^,^36^,^37^.

The electrochemical impedance spectra of all catalysts for ethylene glycol are shown in Fig. S6. The impedance of Pd/NG and PdSn/G is lower than that of the Pd/G catalysts in the case of ethylene glycol oxidation. Furthermore, the impedance of PdSn/NG is lower than that of the Pd/NG and PdSn/G catalysts, indicating the lowest electrochemical polarization impedance. In the low-frequency range, peculiar diffusion control impedance appears for PdSn/NG, which suggests the electrochemical reaction is the fastest among all catalysts. Consequently, the PdSn/NG catalyst presents a lowest charge-transfer resistance compared to other catalysts during ethylene glycol oxidation.

Tafel plots of the as-prepared catalysts were performed at the scan rate of 2 mV s^−1^ in 1 M KOH + 0.5 M (CH_2_OH)_2_ solution, as presented in Fig. S7. All curves contain can be fitted by straight lines with different slopes. The straight line at the given potential region involves ethylene glycol adsorption and dehydrogenation reactions as well as oxidative removal of CO-like species^35^. The Tafel slope values are 0.125, 0.143, 0.123 and 0.137 V dec^−1^ for Pd/G, Pd/NG, PdSn/G and PdSn/NG, respectively. This variation trend in slope value indicates that ethylene glycol dehydrogenation reaction becomes fast and the removal of strongly adsorbed species becomes easy as Sn and N adding into the Pd catalyst.

CO stripping test was employed to evaluate the capability of the catalyst in removal of adsorbed CO-like species. CO stripping curves of Pd/G, Pd/NG PdSn/G and PdSn/NG in 1M KOH solution are shown in Fig. 4. The corresponding peak potentials on the PdSn/NG, PdSn/G, Pd/NG and Pd/G catalysts are about −0.149, −0.135, −0.126 and −0.113 V, respectively. The onset potentials for CO oxidation are about −0.223, −0.201, −0.183 and −0.171 V, respectively. These results suggest that NG support and Sn addition may promote tolerance towards CO_ad_ poisoning of the catalyst. The altered electronic structure of Pd by N and Sn is responsible for this enhanced anti-poisoning ability probably via suppressing the CO adsorption on Pd surface^36^,^37^. PdSn/NG and Pd/NG show an overlap of two peaks in −0.14 ~ −0.06 V, indicating that the two kinds of adsorption states of CO_ad_ on their surface^38^,^39^. PdSn/G and PdSn/NG exhibits the smaller peak area for CO_ad_ oxidation compared to the Pd/G and Pd/NG catalysts because Sn occupies parts of Pd active sites, which is in agreement with the result of hydrogen desorption curves in Fig. S4. Highest durability of PdSn/NG is probably attributed to better stability arised from N-doped graphene supporting environment and the Sn loading on Pd nanoparticle surface. Finally, the electrochemical active area (EAS) of the Pd-based catalysts was calculated based on the CO stripping test as shown in supporting information, which is agree with the result of our former reports^24^.

To further confirm the multisource synergy effect exists in Pd-based catalysts during catalyzing alcohol oxidation, PdPb/NG was additionally prepared with same method and various alcohol electrooxidation activities are also investigated. In Figs 5, S5 and S8–S14, it shows the cyclic voltammograms of the series of Pd-based catalysts with metal alloying and/or (N-doped) graphene in the 1 M KOH + 1 M methanol/1 M ethanol/ 0.5 M ethylene glycol solution. No matter what kind of alcohols was used, PdPb/NG and PdSn/NG show the enhanced catalytic activity and multisource synergetic catalytic effect than just one of metal alloying or N-doped graphene in oxidation of small organic molecules. It concludes that the multisource synergistic catalytic effect is universal in Pd-based anode catalyst during oxidation of various alcohols.

**Figure 6:**
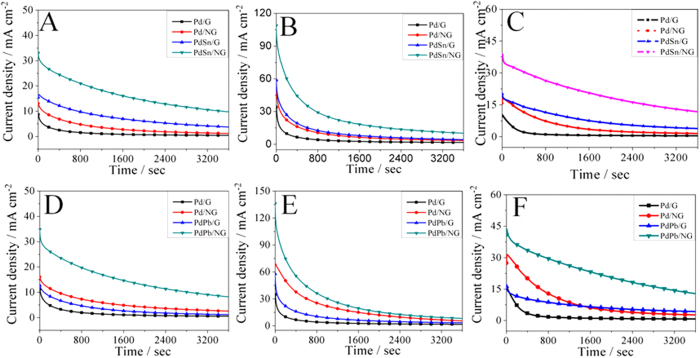
Current–time curves of Pd/G, Pd/NG, PdSn/G and PdSn/NG in 1 M KOH + 1 M methanol(**A**)/1 M ethanol (**B**)/0.5 M ethylene glycol (**C**) solution at the potential of –0.15 V; Current–time curves of Pd/G, Pd/NG, PdPb/G and PdPb/NG in 1 M KOH + 1 M methanol (**A**)/1 M ethanol (**B**)/0.5 M ethylene glycol (**C**) solution at the potential of –0.15 V.

Catalytic durability and stability of Pd/G, Pd/NG, PdSn/G and PdSn/NG are investigated by constant potential tests in 1 M KOH + 1 M methanol (A)/1 M ethanol (B)/0.5 M ethylene glycol (C) solution, as depicted in Fig. 6A–C. Catalytic durability and stability of Pd/G, Pd/NG, PdPb/G and PdPb/NG are investigated by constant potential tests in 1 M KOH + 1 M methanol (D)/1 M ethanol (E)/0.5 M ethylene glycol (F) solution, as depicted in Fig. 6D–F. The current densities of PdSn/NG and PdPb/NG catalysts fall slowly at all stage with high current densities, while the current densities of PdSn/NG and PdPb/NG catalysts decrease slowly at all or part stage with low current densities. The current density of PdSn/NG and PdPb/NG keeps highest in the whole stage among all catalysts and follows the rule and order^12^,^27^. Obviously, the synergetic catalytic effects of metal alloying and N-doped graphene support in the catalytic durability and stability is also revealed.

In summary, a series of Pd-based catalysts with metal alloying (Sn, Pb) and (N-doped) graphene with regular enhanced electrocatalytic activity were demonstrated. Significantly, the PdSn(2:1)/NG catalyst exhibited the highest peak current density of 118.05 mA cm^−2^ on EG oxidation reaction, which is higher than the sum of current densities (45.63 + 47.59 mA cm^−2^) of Pd/NG and PdSn/G. This multisource synergetic catalytic effect of metal (Sn, Pb) alloying and N-doping grapehene support was demonstrated in the the series PdM(M = Sn, Pb)/N-doped graphene nanocomposite during small organic molecule (methanol, ehtanol and Ethylene glycol) oxidation reaction. The high dispersion of small nanoparticles, the altered electron cloud density and Pd(0)/Pd(II) ratio of Pd on the catalyst by strong coupled the metal (Sn, Pb) alloying and N-doped graphene are responsible for the multisource synergistic catalytic effect of PdM(M = Sn, Pb)/NG. On this basis, the PdM(M = Sn, Pb)/NG also showed the enhanced catalytic durability and anti-poisoning. Our results support a new concept to explore new anode catalyst in fuel cell and are applicable to the design of other catalysts for direct alcohols fuel cells (DAFCs).

## Methods

### Sample preparation

#### Chemicals

All analytically pure reagents were used as received without any further purification, and all solutions were prepared with double-distilled water. The support preparation was as follows:

#### Preparation of Graphite oxide (GO)

Graphite oxide (GO) was prepared according to the previous literature by Hummers^40^ from graphite powder (Aldrich, powder, <20 micron, synthetic)^41^. N-doped graphene was further prepared from as-prepared graphite oxide and the typical experiment procedure was as follows: N-doped graphene was synthesized by adding appropriate amount of graphite oxide (GO), double-distilled water and ammonium hydroxide under ultrasonic stirring for 90 min. Then, the well-dispersed suspension solution was transferred into a Teflon bottle of 80 mL held in a stainless steel autoclave, which was filled up to the capacity of 75%, sealed and maintained at 200 °C for 6 h. After the autoclave had been cooled down to room temperature naturally, the precipitate was collected and washed repeatedly with double-distilled water, and then dried in the freezer dryer at –55 °C for 24 h under vacuum. After suitable grinding, the mixture was annealed under argon atmosphere at 600 °C for 4 h. The final material was obtained after cooling to room temperature in the furnace. The same method without ammonium hydroxide was used to prepare graphene (G).

#### Preparation of all these catalysts

The PdSn/NG catalyst was synthesized as follows: appropriate amount of N-doped graphene, 8 ml of 18.9 mM PdCl_2_ and 75.6 mM HCl aqueous solution were mixed with 70 ml of ethylene glycol (EG) in a flask under ultrasonic stirring. Then, a specific volume of 18.9 mM SnCl_2_ ethylene glycol solution was then added dropwise to the suspension with constant stirring for 1 h. Then, 80 mg of NaBH_4_ was dissolved in 70 ml of water and this solution was added dropwise into the suspension above to reduce Pd^2+^ and Sn^2+^ ions. This redox reaction was conducted for 2 h. Then the suspension was filtered, washed with double-distilled water and dried in a vacuum oven overnight to obtain the PdSn/NG composite. The similar synthetic method was used to obtain the Pd/G, Pd/NG, PdSn/G, PdSn/NG catalyst with different Pd/Sn atomic ratios. The total Pd metal loading of all these catalysts was maintained at 15 wt.%. For optimizing, the Pd/G, Pd/NG, PdPb/G and PdPb/NG catalysts with different Pd/Pb atomic ratios were synthesized according to the same procedure. The total Pd metal loading of all these catalysts was maintained at 20 wt.%.

### Measurements

The Pd loadings and atomic ratios in the samples were determined using an Ultima2 inductively coupled plasma OES spectrometer (ICP-OES, Jobin Yvon). Before the tests, carbon in the samples was removed at 700 °C; then the residue was dissolved in a mixed solution containing 5 ml HF, 4 ml HNO_3_ and a few drops of HClO_4_. This process was repeated twice after acid evaporation. Finally, the residue was dissolved in chloroazotic acid and the mixture was heated until dryness. An aqueous HNO_3_ solution was then added and the solution was transferred to a volumetric flask for analysis. The dispersion of the catalyst was characterized using a JEOL JEM-1010 transmission electron microscope (TEM) at 200 kV. The X-ray powder diffraction (XRD) was conducted using a Philip X’Pert Pro MPP X-ray powder diffractometer with Cu Kα radiation (λ = 1.54 Å) at the scan rate of 5° min^–1^ with a step of 0.02°. The chemical valences of elements in the catalyst were analyzed by X-ray photoelectron spectroscopy (XPS, VG ESCALAB 250) with an Al Kα X-ray source of 1487 eV. The chamber pressure was kept below 3 × 10^−10^ mbar during test and specific correction was conducted by using a C 1s binding energy of 285 eV.

Electrochemical measurements were conducted by using a CHI660E electrochemical working station (CH Instrument Inc.). A mercuric oxide electrode (Hg/HgO/1 M KOH, 0.098 V vs. SHE^42^) and a Pt rod were used as the reference and counter electrodes, respectively. A piece of glassy carbon (0.1256 cm^2^) covered by the catalyst was used as the working electrode. For the working electrode preparation, a specific amount of the catalyst was dispersed in a suspension of 45 μl of 20 wt.% nafion solution (DuPont, USA) and 1955 μl of isopropyl alcohol under ultrasonic stirring. A 4 μl aliquot of the slurry was spread on the glassy carbon surface which was first polished with Al_2_O_3_ paste, treated in an ultrasonic bath and then rinsed by double-distilled water and anhydrous ethanol in sequence. The electrode was dried at 60 °C for 25 min. The total Pd loading on the electrode was kept at 4 μg. Electrochemical impedance spectra in 1 M KOH + 0.5 M (CH_2_OH)_2_ solution at −0.15 V. The amplitude of the modulation potential is 5 mV. The frequency ranged from 100 kHz to 100 mHz; All solutions were first de-aerated with high purity N_2_ before measurements. All electrochemical measurements were conducted in a thermostatic water bath at 30 °C.

It is known that the electrochemical active surface area (EAS) of the catalysts can be determined by electrochemical oxidation of a pre–adsorbed saturated COads layer (COads stripping) and subsequent base voltammetry (1 cycle) in CO–free supporting electrolyte. The corresponding EAS of the catalyst can be calculated based on the following equation:


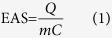



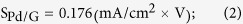



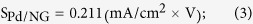



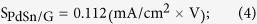



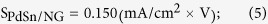



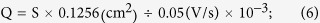



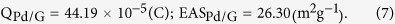



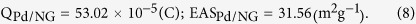



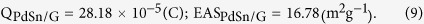



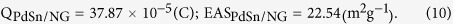


Where Q is the charge for CO desorption–electrooxidation, m is the amount of Pd loaded, and C (420 μC cm^−2^) is the charge needed for the adsorption of a CO monolayer. The calculated EAS for Pd/G, Pd/NG, PdSn/G and PdSn/NG is 26.3, 32.6, 16.8 and 22.5 m^2^ g^−1^.

## Additional Information

**How to cite this article**: Wu, P. *et al*. Multisource Synergistic Electrocatalytic Oxidation Effect of Strongly Coupled PdM (M = Sn, Pb)/N-doped Graphene Nanocomposite on Small Organic Molecules. *Sci. Rep*. **5**, 14173; doi: 10.1038/srep14173 (2015).

## Supplementary Material

Supplementary Information
