# Supplementary material for: Multisource Synergistic Electrocatalytic Oxidation Effect of Strongly Coupled PdM (M = Sn, Pb)/N-doped Graphene Nanocomposite on Small Organic Molecules
Source: Sci Rep. 2015 Oct 5;5:14173. doi: 10.1038/srep14173 (PMC4593028; doi:10.1038/srep14173)
Supplement: Supplementary Information [file srep14173-s1.pdf]

# Multisource Synergistic Electrocatalytic Oxidation Effect of Strongly Coupled PdM (M=Sn, Pb) /N-doped Graphene Nanocomposite on Small Organic Molecules

Peng Wu[a], Yiyin Huang[a], Longtian Kang[a], Maoxiang Wu[a], Yaobing Wang[a,]*

[a] State Key Laboratory of Structural Chemistry; Key Laboratory of design and assembly of functional nanostructures, Fujian Institute of Research on the Structure of Matter, Chinese Academy of Sciences, YangQiao West Road 155#, Fuzhou, 350002, P. R. China. E-mail: wangyb@fjirsm.ac.cn

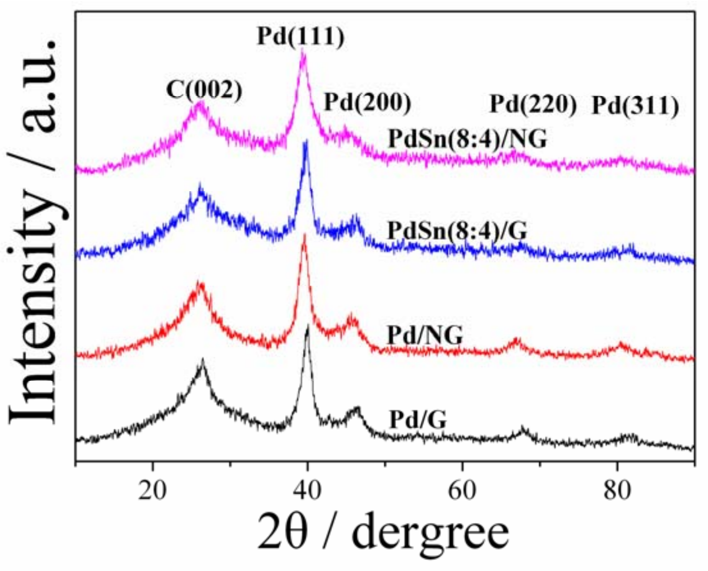


**Figure S1.** XRD patterns of Pd/G, Pd/NG, PdSn(8:4)/NG and PdSn(8:4)/NG.

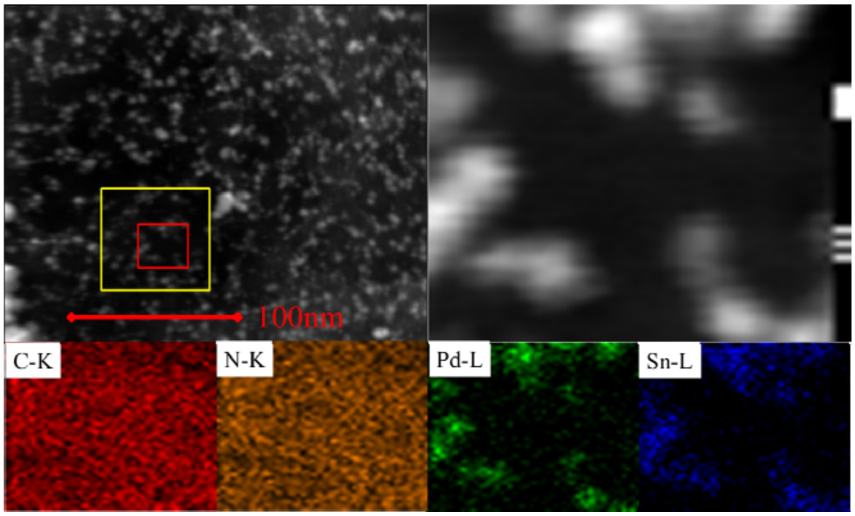


**Figure S2.** EELS mappings of the PdSn/NG sample showing the distributions of C, N, Pd and Sn.

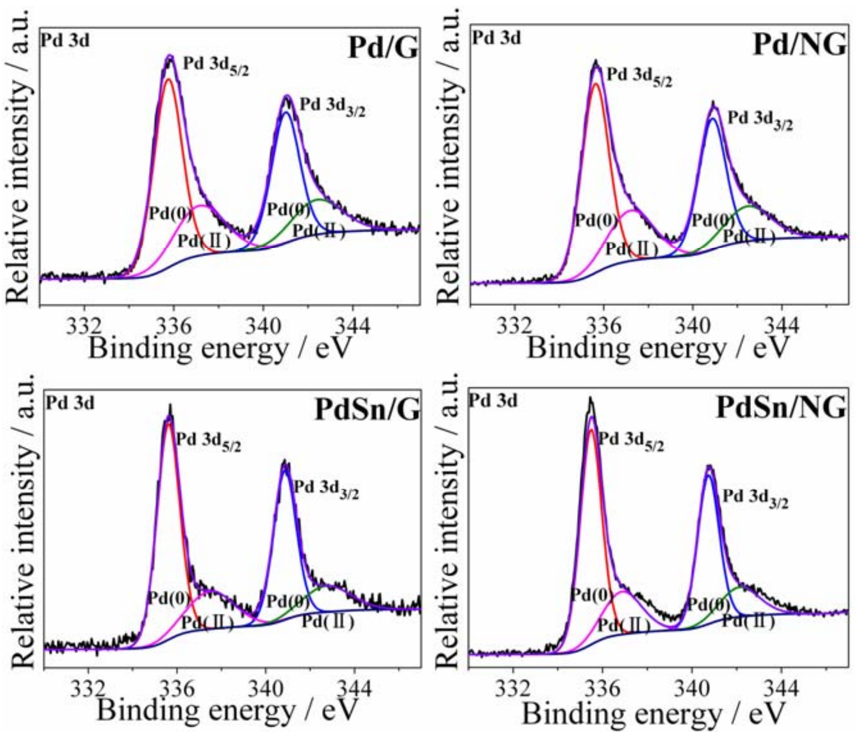


**Figure S3.** XPS spectrum of Pd/G, Pd/NG, PdSn/G and PdSn/NG in Pd 3d region.

**Table S1.** The ratios between bivalent species and metallic Pd.

| ***BEs/eV*** | ***Pd3d$_{5/2}$* [Pd( Ⅱ): Pd(0)] ratio** | ***Pd3d$_{3/2}$* [Pd( Ⅱ):Pd(0)] ratio** |
|---|---|---|
| ***Pd/G*** | **0.54:1** | **0.54:1** |
| ***Pd/NG*** | **0.49:1** | **0.50:1** |
| ***PdSn/G*** | **0.40:1** | **0.43:1** |
| ***PdSn/NG*** | **0.40:1** | **0.41:1** |

**Table S2.** The peak of XPS spectrum in Pd 3d region

| ***BEs/eV*** | ***Pd3d$_{5/2}$*** | ***Pd3d$_{3/2}$*** |
|---|---|---|
| ***Pd/G*** | **335.8** | **341.0** |
| ***Pd/NG*** | **335.7** | **340.9** |
| ***PdSn/G*** | **335.7** | **340.8** |
| ***PdSn/NG*** | **335.5** | **340.7** |

**Table S3.** The peak of XPS spectrum in Sn 3d region

| ***BEs/eV*** | ***Sn3d$_{5/2}$*** | ***Sn3d$_{3/2}$*** |
|---|---|---|
| ***PdSn/G*** | **486.6** | **495.1** |
| ***PdSn/NG*** | **487.0** | **495.4** |

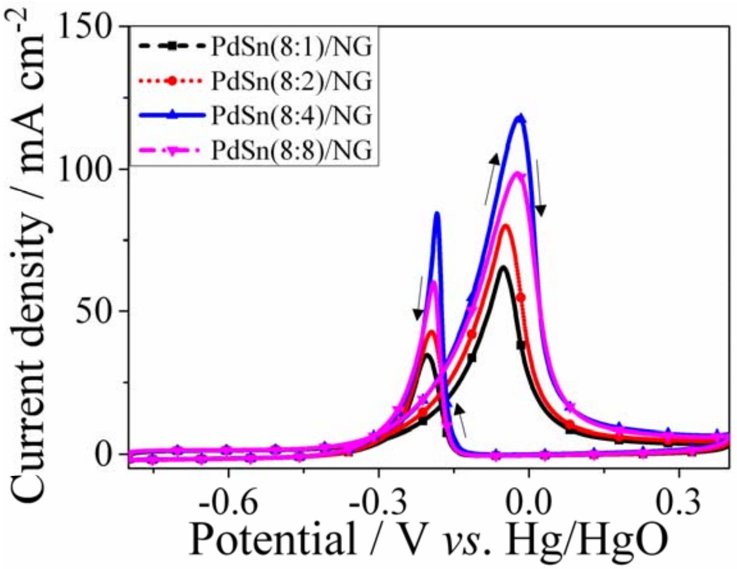


**Figure S4.** Cyclic voltammograms of PdSn/NG with different Pd/Sn atomic ratios catalysts in the 1 M KOH + 0.5 M $(CH_2OH)_2$ solution. Scan rate: 50 mV $s^{-1}$.

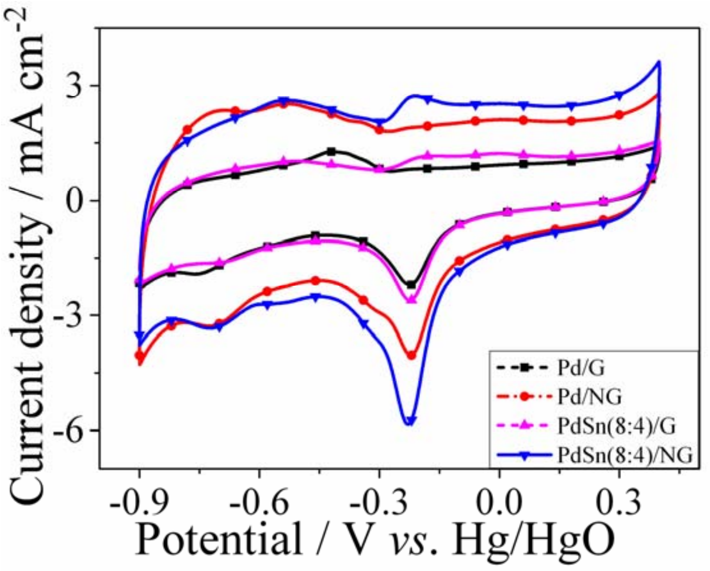


**Figure S5.** Cyclic voltammograms of Pd/G, Pd/NG, PdSn(2:1)/G and PdSn(2:1)/NG in the 1 M KOH solution. Scan rate: 50 mV $s^{-1}$.

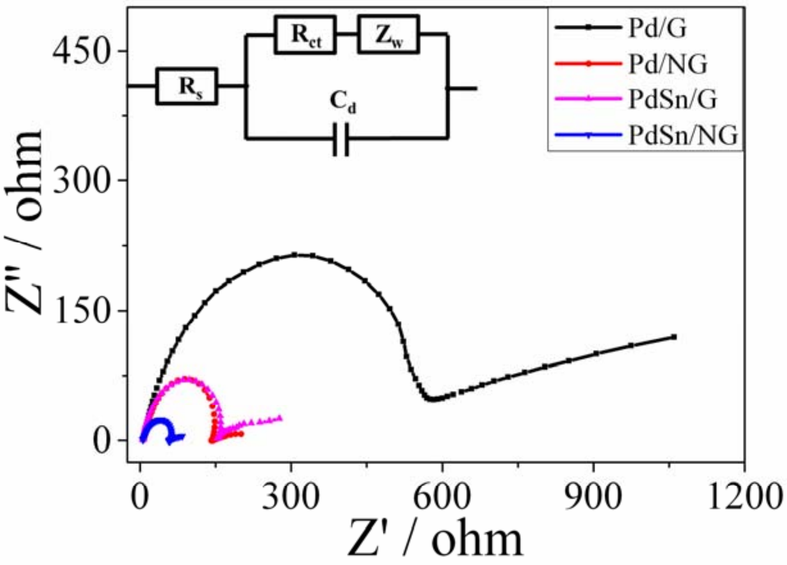


**Figure S6.** Electrochemical impedance spectra in 1 M KOH + 0.5 M $(CH_2OH)_2$ solution at −0.15 V. The amplitude of the modulation potential is 5 mV. The frequency ranged from 100 kHz to 100 mHz and the equivalent circuit used to fit the impedance spectra.

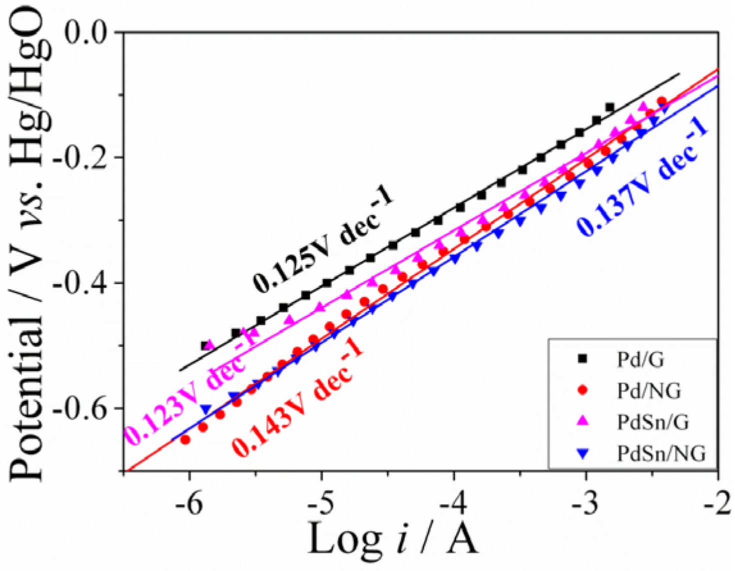


**Figure S7.** Tafel plots of the as-prepared catalysts were performed at the scan rate of 2 mV $s^{-1}$ in 1 M KOH + 0.5 M $(CH_2OH)_2$ solution.

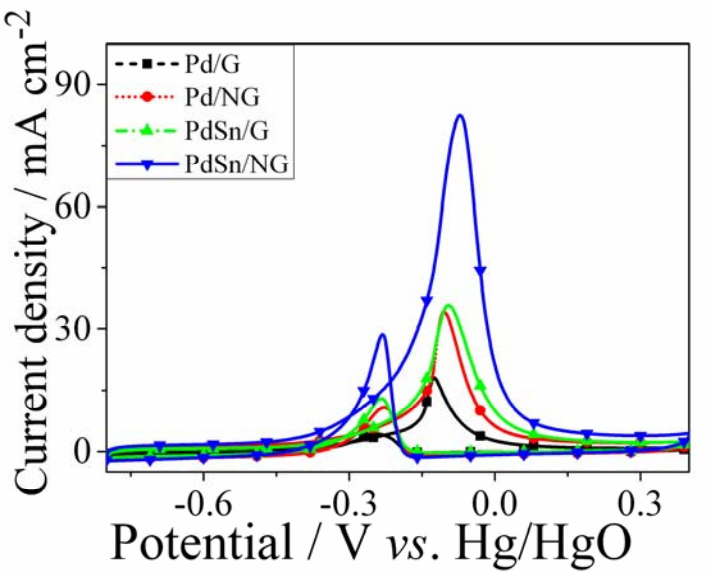


**Figure S8.** Cyclic voltammograms of Pd/G, Pd/NG, PdSn/G and PdSn/NG in the 1 M KOH + 0.5 M $CH_3OH$ solution. Scan rate: 50 mV $s^{-1}$.

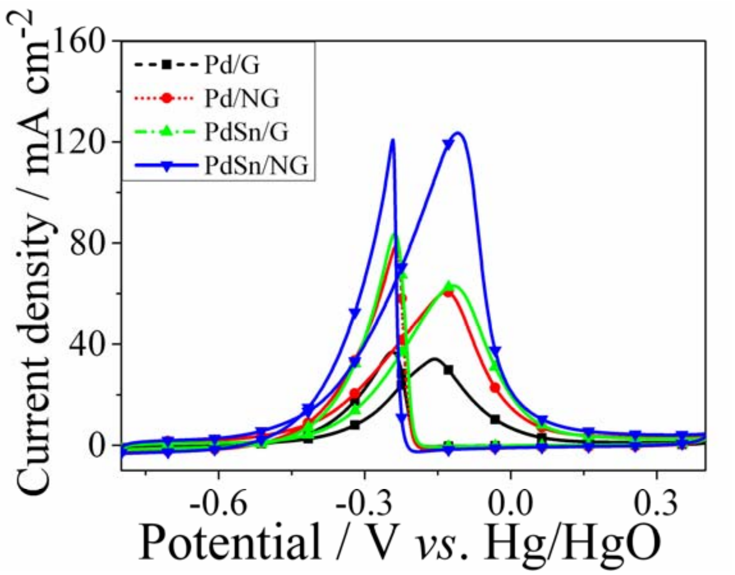


**Figure S9.** Cyclic voltammograms of Pd/G, Pd/NG, PdSn/G and PdSn/NG in the 1 M KOH + 0.5 M $C_2H_5OH$ solution. Scan rate: 50 mV $s^{-1}$.

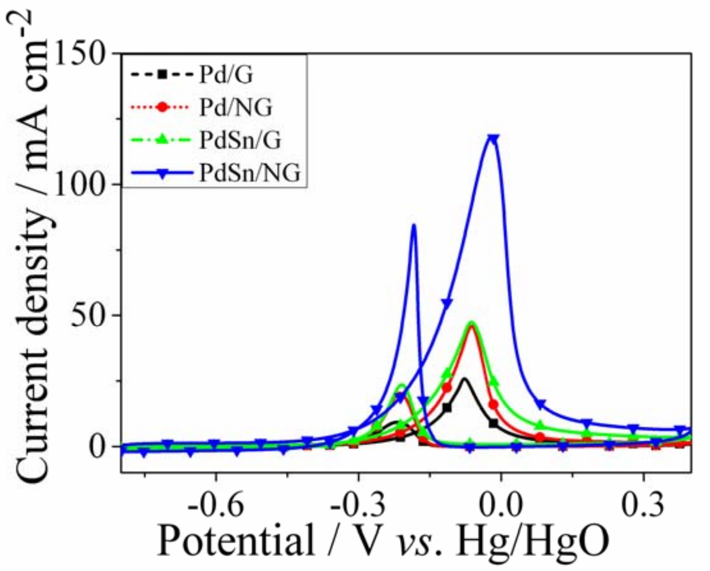


**Figure S10.** Cyclic voltammograms of Pd/G, Pd/NG, PdSn/G and PdSn/NG in the 1 M KOH + 0.5 M $(CH_2OH)_2$ solution. Scan rate: 50 mV $s^{-1}$.

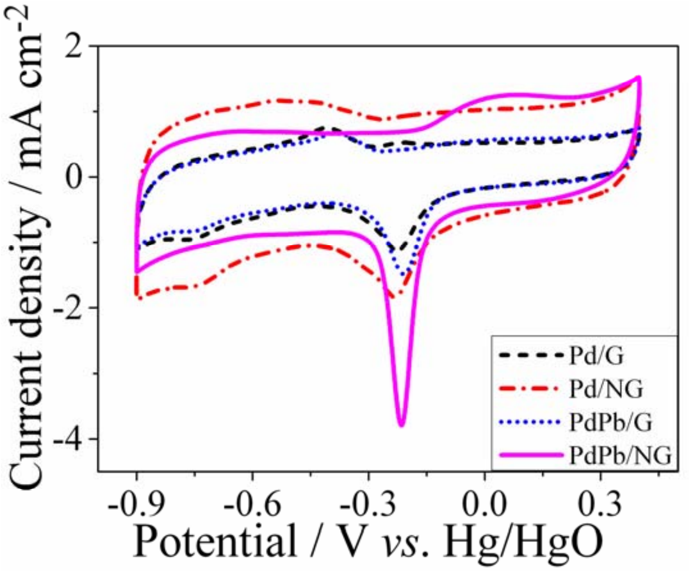


**Figure S11.** Cyclic voltammograms of Pd/G, Pd/NG, PdPb(2:1)/G and PdPb(2:1)/NG in the 1 M KOH solution. Scan rate: 50 mV $s^{-1}$.

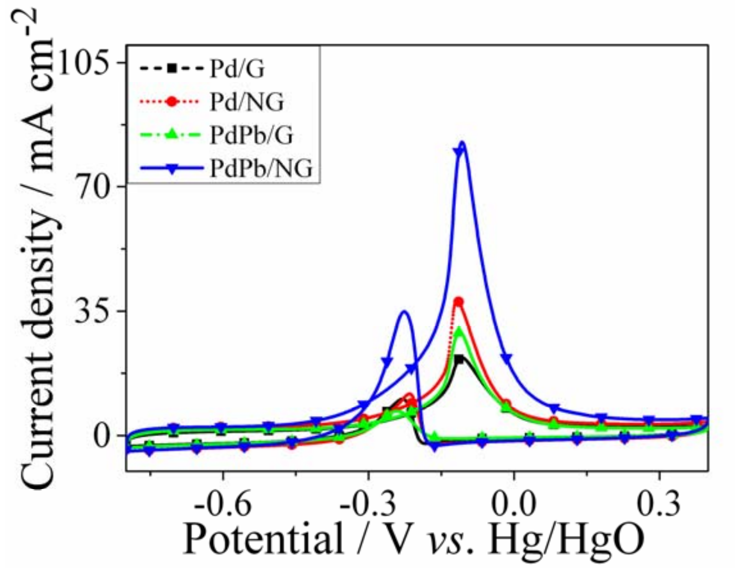


**Figure S12.** Cyclic voltammograms of Pd/G, Pd/NG, PdPb/G and PdPb/NG in the 1 M KOH + 0.5 M $CH_3OH$ solution. Scan rate: 50 mV $s^{-1}$.

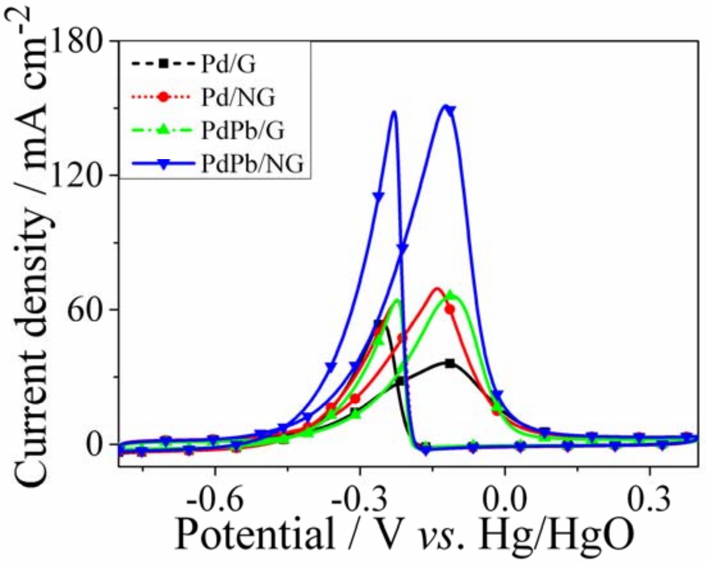


**Figure S13.** Cyclic voltammograms of Pd/G, Pd/NG, PdPb/G and PdPb/NG in the 1 M KOH + 0.5 M $C_2H_5OH$ solution. Scan rate: 50 mV $s^{-1}$.

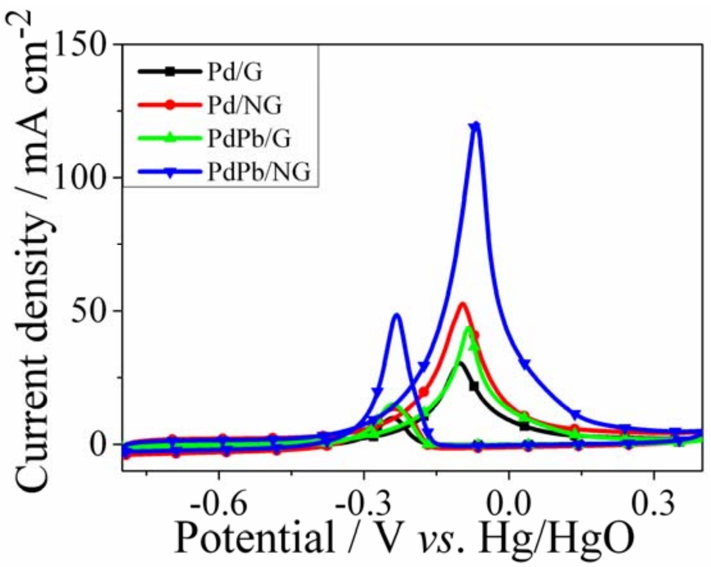


**Figure S14.** Cyclic voltammograms of Pd/G, Pd/NG, PdPb/G and PdPb/NG in the 1 M KOH + 0.5 M $(CH_2OH)_2$ solution. Scan rate: 50 mV $s^{-1}$.
